# Association of Preterm Birth with Inflammatory Bowel Disease and Salivary Gland Disease: Machine Learning Analysis Using National Health Insurance Data

**DOI:** 10.3390/ijerph19053056

**Published:** 2022-03-05

**Authors:** Kwang-Sig Lee, Eun Sun Kim, In-Seok Song, Hae-In Kim, Ki Hoon Ahn

**Affiliations:** 1 AI Center, Korea University Anam Hospital, Seoul 02841, Korea; ecophy@hanmail.net (K.-S.L.); radis13@korea.ac.kr (H.-I.K.); 2 Department of Gastroenterology, Korea University Anam Hospital, Seoul 02841, Korea; silverkes@naver.com; 3 Department of Oral and Maxillofacial Surgery, Korea University Anam Hospital, Seoul 02841, Korea; sis80@naver.com; 4 School of Industrial Management Engineering, Korea University, Seoul 02841, Korea; 5 Department of Obstetrics and Gynecology, Korea University Anam Hospital, Seoul 02841, Korea

**Keywords:** preterm birth, inflammatory bowel disease, ulcerative colitis, Crohn’s disease, salivary gland disease, proton pump inhibitors

## Abstract

This study employs machine learning and population data for testing the associations of preterm birth with inflammatory bowel disease (IBD), salivary gland disease, socioeconomic status and medication history, including proton pump inhibitors. The source of population-based retrospective cohort data was the Korea National Health Insurance Service claims data for all women aged 25–40 years and who experience their first childbirths as singleton pregnancy during 2015 to 2017 (402,092 women). These participants were divided into the Ulcerative Colitis (UC) Group (1782 women), the Crohn Group (1954 women) and the Non-IBD Group (398,219 women). For each group, the dependent variable was preterm birth during 2015–2017, and 51 independent variables were included. Random forest variable importance was employed for investigating the main factors of preterm birth and testing its associations with salivary gland disease, socioeconomic status and medication history for each group. The proportion of preterm birth was higher for the UC Group and the Non-IBD Group than for the Crohn Group: 7.86%, 7.17% vs. 6.76%. Based on random forest variable importance, salivary gland disease was a top 10 determinant for the prediction of preterm birth for the UC Group, but this was not the case for the Crohn Group or the Non-IBD Group. The top 5 variables of preterm birth for the UC Group during 2015–2017 were socioeconomic status (8.58), age (8.00), proton pump inhibitors (2.35), progesterone (2.13) and salivary gland disease in 2014 (1.72). In conclusion, preterm birth has strong associations with ulcerative colitis, salivary gland disease, socioeconomic status and medication history including proton pump inhibitors.

## 1. Introduction

Preterm birth and inflammatory bowel disease (IBD) are major parts of the disease burden on the globe [1,2,3,4,5,6,7,8,9]. Every year, 15 million babies are born preterm in the world, and preterm birth is a main contributor for global neonatal and childhood mortality, i.e., 1 million deaths among those aged 0 to 4 years [1,2]. For example, 1 out of every 10 babies was preterm in the United States during 2003–2012, that is, 5,042,982 (12.2%) of 41,206,315 newborns [3]. Cost-effective interventions are expected to prevent three-quarters of mortality from preterm birth [4]. During 1990–2017, indeed, the global prevalence of IBD registered a rapid growth of 85.1% from 3.7 million to 6.8 million. IBD (or “non-infectious chronic inflammation of the gastrointestinal tract”) includes ulcerative colitis (UC) and Crohn’s disease. Its precise etiology is still unknown, but it is assumed to result from an inappropriate immune response to environmental factors such as microbial antigens in genetically susceptible hosts [5]. IBD is common among young reproductive-aged women, and it is reported to cause salivary problems with the lack of antimicrobial peptides [6,7,8,9]. In this context, it is a plausible hypothesis that preterm birth has strong associations with IBD and salivary gland disease.

To our best knowledge, however, no study with population-based data has been available on the associations of preterm birth with IBD and salivary gland disease. This study presents a comprehensive machine-learning analysis on this topic, using a population-based cohort of 402,092 participants and a rich collection of 51 predictors. This study brings new results on the associations of preterm birth with UC, salivary gland disease, socioeconomic status and medication history, including proton pump inhibitors. Proton pump inhibitors medication, which is common for gastrointestinal disease, is expected to cause gastric acidity and compositional changes in the gut microbiota [10]. It is a plausible hypothesis that these changes can lead to the increased risks of infection, IBD, salivary gland disease and preterm birth.

## 2. Materials and Methods

### 2.1. Participants and Variables

The source of retrospective cohort data for this study was the Korea National Health Insurance Service claims data for all women in Korea who aged 25–40 years and gave first childbirths as singleton pregnancy during 2015 to 2017 (402,092 women). These inclusion criteria were adopted given that data with a wider range and a bigger size went beyond the maximum capacity of the Korea National Health Insurance Service data analysis center located in Seoul, Korea.

The 402,092 participants were divided into the UC Group (1782 women), the Crohn Group (or Crohn’s Disease Group) (1954 women) and the Non-IBD Group (398,219 women). For each group, the dependent variable was preterm birth during 2015–2017 (birth before 37 weeks of gestation). Four categories of preterm birth were introduced according to the ICD-10 Code: (1) PTB 1—preterm birth with premature rupture of membranes (PROM) only; (2) PTB 2—preterm labor and birth without PROM; (3) PTB 3—PTB 1 or PTB 2; (4) PTB 4—PTB 3 or other indicated preterm birth (Table S1, a supplementary table). Each of these categories was coded as “no” vs. “yes”. A total of 51 independent variables covered the following information: (1) demographic/socioeconomic determinants in 2014 including age (years), socioeconomic status measured by an insurance fee with the range of 1 (the highest group) to 20 (the lowest group), and region (city) (no vs. yes); (2) disease information (no vs. yes) for each of the years 2002–2014, namely, diabetes, hypertension and salivary gland disease; (3) medication history (no vs. yes) in 2014, that is, benzodiazepine, calcium channel blocker, nitrate, progesterone, proton pump inhibitor, sleeping pills and antidepressant [10,11,12]; (4) obstetric information (no vs. yes) in 2014 including in vitro fertilization, myoma uteri and prior cone. The 39 disease variables were presented as Diabetes_2002, …, Diabetes_2014, Hypertension_2002, …, Hypertension_2014, and Salivary_Gland_2002, …, Salivary_Gland_2014. The disease information and the medication history were screened from ICD-10 and ATC codes, respectively (Tables S1 and S2, supplementary tables). Here, the definition of diabetes was based on fasting glucose equal to or higher than 126 mg/dL or antidiabetic medication [13], while the definition of hypertension was based on systolic/diastolic blood pressure equal to or higher than 140/90 mmHg or antihypertensive medication [14]. The definitions of UC, Crohn’s disease and salivary gland disease were adopted from the Mayo Clinic and the National Institute of Health: (UC) “an inflammatory bowel disease causing the inflammations and ulcers (sores) of the digestive tract” (https://www.mayoclinic.org/diseases-conditions/ulcerative-colitis/symptoms-causes/syc-20353326) (accessed on 1 December 2021); (Crohn’s disease) “an inflammatory bowel disease causing the inflammations of the digestive tract, which can lead to abdominal pain, severe diarrhea, fatigue, weight loss and malnutrition” (https://www.mayoclinic.org/diseases-conditions/crohns-disease/symptoms-causes/syc-20353304) accessed on (1 December 2021); (salivary gland disease) “if the salivary glands are damaged or aren’t producing enough saliva, it can affect taste, make chewing and swallowing more difficult, and increase the risk for cavities, tooth loss, and infections in the mouth” (https://www.nidcr.nih.gov/health-info/saliva-salivary-gland-disorders) accessed on (1 December 2021).

### 2.2. Analysis

Logistic regression, the random forest and the artificial neural network were employed for the prediction of preterm birth [10,11,12]. The three models were chosen based on the results of a recent review on the application of artificial intelligence in early diagnosis of preterm birth [11]: The summary of the review suggests that different machine learning approaches would be optimal for different types of data regarding the prediction of preterm birth, that is, logistic regression, random forest and/or the artificial neural network for numeric data. The number of trees was 100, and GINI was adopted as node impurity for the random forest. The number of hidden layers was 5 and the Broyden-Fletcher-Goldfarb-Shanno algorithm was used as an optimization algorithm for the artificial neural network. The data of 402,092 cases were split into training and validation sets with a 70:30 ratio (281,464 vs. 120,628 cases). A criterion for the validation of the trained models was accuracy, a ratio of correct predictions among 120,628 cases. Random forest variable importance (total decrease in GINI averaged over 100 trees) was introduced for investigating the main factors of preterm birth and testing its associations with salivary gland disease, socioeconomic status and medication history including proton pump inhibitors. R-Studio 1.3.959 (R-Studio Inc.: Boston, MA, USA) was employed for the analysis from 1 August 2020 to 31 January 2021.

This retrospective study was approved by the Institutional Review Board (IRB) of Korea University Anam Hospital, Seoul, Korea, on 5 November 2018 (2018AN0365). Informed consent was waived by the IRB.

## 3. Results

### 3.1. Descriptive Statistics

**Figure 1:**
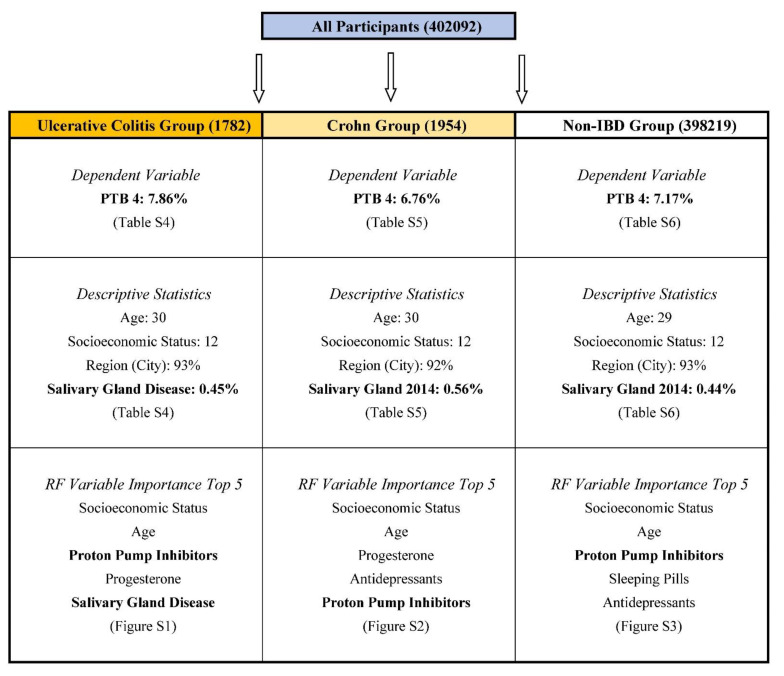
Summary of Results. Important variables are highlighted as bold. The proportion of preterm birth was higher for the Ulcerative Colitis (UC) Group and the Non-Inflammatory-Bowel-Disease (Non-IBD) Group than for the Crohn Group: 7.86%, 7.17% vs. 6.76%. Based on random forest variable importance, salivary gland disease was a top 10 determinant for the prediction of preterm birth for the UC Group, but it was not the case for the Crohn Group or the Non-IBD Group. The top 5 variables of preterm birth for the UC Group during 2015–2017 were socioeconomic status (8.58), age (8.00), proton pump inhibitors (2.35), progesterone (2.13) and salivary gland disease in 2014 (1.72). Note: IBD Inflammatory Bowel Disease, PTB Preterm Birth, RF Random Forest.

The results of this study are summarized in Figure 1. Descriptive statistics for participants’ preterm birth and its determinants are shown in Tables S3–S6 (Supplementary Tables), i.e., all participants (Table S3), the UC Group (Table S4), the Crohn Group (Table S5) and Non-IBD Group (Table S6). The proportion of preterm birth was higher for the UC Group and the Non-IBD Group than for the Crohn Group: (1) 7.52%, 6.90% vs. 6.60% (PTB 3); (2) 7.86%, 7.17% vs. 6.76% (PTB 4); (3) 0.34%, 0.27% vs. 0.16% (PTB 4 excluded in PTB 3). Indeed, the proportion of salivary gland disease in 2014 was higher for the UC Group and the Crohn Group than for the Non-IBD Group (0.45% and 0.56% vs. 0.41%).

### 3.2. Random Forest Variable Importance

In terms of accuracy, the random forest was slightly better than logistic regression and the artificial neural network (Table 1). The average values of random forest variable importance for PTB 1, PTB 2, PTB 3 and PTB 4 are presented in Figures S1–S3 (Supplementary Figures), i.e., the UC Group (Figure S1), the Crohn Group (Figure S2) and Non-IBD Group (Figure S3). Based on the average values of random forest variable importance for PTB 1, PTB 2, PTB 3 and PTB 4, salivary gland disease was a top 10 determinant for the prediction of preterm birth for the UC Group, but it was not the case for the Crohn Group or the Non-IBD Group. The top 10 variables of preterm birth for the UC Group during 2015–2017 in Figure S1 were socioeconomic status (8.58), age (8.00), proton pump inhibitors (2.35), progesterone (2.13), salivary gland disease in 2014 (1.72), region (1.69), antidepressants (1.66), sleeping pills (97.23), benzodiazepine (1.20) and nitrate (1.01). The top 10 variables of preterm birth for the Crohn Group during 2015–2017 in Figure S2 were socioeconomic status (8.09), age (6.56), progesterone (2.02), antidepressants (1.67), proton pump inhibitors (1.64), diabetes in 2011 (1.46), benzodiazepine (1.44), sleeping pills (1.31), diabetes in 2014 (1.20) and myoma uteri (1.09). Likewise, the top 10 variables of preterm birth for the Non-IBD Group during 2015–2017 in Figure S3 were socioeconomic status (362.97), age (326.77), proton pump inhibitors (61.47), sleeping pills (51.92), antidepressants (50.39), region (48.85), progesterone (48.59), benzodiazepine (43.16), calcium channel blockers (31.93) and diabetes in 2013 (31.92). These were the average values for PTB1, PTB 2, PTB 3 and PTB 4.

### 3.3. Logistic Regression Coefficient Estimated

The results of logistic regression (Table 2, Table 3 and Table 4) present the sign and magnitude for the effect of a major determinant on preterm birth, i.e., the UC Group (Table 2), the Crohn Group (Table 3) and the Non-IBD Group (Table 4). For instance, the odds of PTB 4 in the UC Group will be greater by 4355% for those with salivary gland disease in 2014 than those without it (Table 2).

## 4. Discussion

### 4.1. Findings of Study

In general, the proportion of preterm birth was higher for the UC Group and the Non-IBD Group than for the Crohn Group. Based on random forest variable importance, salivary gland disease was a top 10 determinant for the prediction of preterm birth for the UC Group, but it was not the case for the Crohn Group or the Non-IBD Group. The top 5 variables of preterm birth for the UC Group during 2015–2017 were socioeconomic status, age, proton pump inhibitors, progesterone and salivary gland disease in 2014.

### 4.2. Contributions of Study

Existing literature focuses on microbial infection as a possible mechanism between IBD and preterm birth or salivary gland disease [15,16,17]. In other words, preterm birth and salivary gland disease can be considered to be these extra-intestinal manifestations of IBD. However, the strength of the association among the three diseases can vary between UC and Crohn’s disease as two types of IBD. For example, preterm birth had a stronger association with UC than with Crohn’s disease in a previous study [16]. This agrees with the result of this study: The proportion of preterm birth was higher for the UC Group and the Non-IBD Group than for the Crohn Group. One possible explanation for this finding is that the Crohn’s Group pays more attention to disease treatment and makes less attempts at pregnancy compared to the UC Group and that this leads to a discrepancy between their proportions of preterm birth. To our best knowledge, however, no machine-learning study with population-based data have been available on this topic. This study presents a comprehensive machine-learning analysis on this topic, using a population-based cohort of 402,092 participants and a rich collection of 51 predictors. Specifically, this study brings new results on an association among proton pump inhibitors medication before pregnancy, UC and preterm birth. A systematic review of 60 studies with 134,940 participants in total reports no significant relationship between proton pump inhibitors, medication during pregnancy and preterm birth [18]. A more recent systematic review of 26 observational studies confirmed this finding [19]. However, this study considered proton pump inhibitors before pregnancy instead of its gestational counterpart. Based on the random forest variable importance of this study for the UC Group, proton pump inhibitors in 2014 (before pregnancy) were the third most important determinant of preterm birth during 2015–2017, second only to socioeconomic status in 2014 and age in 2014 (the average for PTB1, PTB 2, PTB 3 and PTB 4). One possible explanation for this finding is that the use proton pump inhibitors before pregnancy causes gastric acidity and compositional changes in the gut microbiota, which lead to the increased risks of infection, UC and preterm birth [10,20]. The number of predictors and the size of the data in this study exceed those of the existing literature. For this reason, the results of this study would be more robust than those in the previous studies.

### 4.3. Limitations of Study

Firstly, this study did not examine possible mediating effects among variables. Secondly, this study adopted the binary category of preterm birth as no vs. yes. However, preterm birth can have multiple categories, and it will be a good topic for future study to compare different predictors for various categories of preterm birth, e.g., extremely preterm (less than 28 weeks), very preterm (28 to 32 weeks) and moderate to late preterm (32 to 37 weeks). Thirdly, this study considered the most comprehensive collection of diseases and medications regarding preterm birth available in the Korea National Health Insurance Service data, but it was not the scope of this study to explore and evaluate various pathways among diseases, complications, medications and preterm birth. Little research has been conducted, and more investigation is needed on this topic. Fourthly, according to a recent review, optimal machine learning methods are expected to vary with different types of data for predicting preterm birth: the random forest, logistic regression and/or the artificial neural networsk in the case of numeric data; the support vector machine in the case of electrohysterogram data; the convolutional neural network in the case of image data; and the recurrent neural network in the case of text data [11]. Uniting various kinds of machine learning approaches for various kinds of preterm birth data would bring new innovations and deeper insights in this line of research. Fifthly, further investigations of single vs. multiple gestation would deliver more insights and more detailed clinical implications. Sixthly, considering the following variables would extend the horizon of research in this direction much further: nutritional status, working conditions, drinking, smoking, biochemical markers, cervical length, cesarean section, familial predisposition, fetal malformation and uterine malformation. Seventhly, model calibration was not considered in this study. Eighthly, there might be room for improvement in the areas under the receiver operating characteristic curves in this study. Ninthly, errors were likely to exist in the stage of data collection but examining this issue was beyond the scope of this study. Little literature is available and more investigation is required on this topic. Finally, the numbers of participants with PTB 4 excluded in PTB 3 and salivary gland disease in the UC Group and the Crohn Group were very small in this study. The participants for this study aged 25–40 years and gave first childbirths as singleton pregnancy during 2015–2017. These inclusion criteria were adopted, given that data with a wider range and a bigger size went beyond the maximum capacity of the Korea National Health Insurance Service data analysis center located in Seoul, Korea. Expanding the data is expected to improve the validity and reliability of this study further.

## 5. Conclusions

Preterm birth has strong associations with ulcerative colitis, salivary gland disease, socioeconomic status and medication history including proton pump inhibitors. For preventing preterm birth, appropriate medication would be needed alongside preventive measures for ulcerative colitis and salivary gland disease and the promotion of socioeconomic status for pregnant women.

## Figures and Tables

**Table 1:** Model Performance.

Model	*Accuracy*				*AUC* ^a^			
	PTB 1 ^b^	PTB 2	PTB 3	PTB 4	PTB 1	PTB 2	PTB 3	PTB 4
*Ulcerative* *Colitis*								
Logistic Regression	0.9238	0.9704	0.9064	0.9140	0.5783	0.6645	0.5334	0.5117
Artificial Neural Network	0.9390	0.9586	0.9232	0.9215	0.5000	0.5377	0.5000	0.5000
Random Forest	0.9390	0.9724	0.9232	0.9215	0.5444	0.5854	0.5783	0.5551
*Crohn*								
Logistic Regression	0.9478	0.9715	0.9096	0.9216	0.5607	0.6082	0.5142	0.5290
Artificial Neural Network	0.9513	0.9715	0.9334	0.9319	0.5000	0.6276	0.5000	0.5000
Random Forest	0.9513	0.9750	0.9334	0.9319	0.5709	0.5915	0.5946	0.5896
*Non-IBD* ^c^								
Logistic Regression	0.9450	0.9767	0.9308	0.9283	0.5501	0.5905	0.5516	0.5562
Artificial Neural Network	0.9450	0.9767	0.9308	0.9283	0.5350	0.5873	0.5000	0.5558
Random Forest	0.9450	0.9767	0.9308	0.9283	0.5092	0.5161	0.5110	0.5153

Note. ^a^ Area Under the Receiver Operating Characteristic Curve. ^b^ PTB Preterm Birth during 2015–2017. ^c^ IBD Inflammatory Bowel Disease.

**Table 2:** Logistic Regression Results for Each Type of Preterm Birth: Ulcerative Colitis Group.

Determinant	*PTB 1* ^a^	*PTB 2*	*PTB 3*	*PTB 4*
Age	1.0098	0.9382	1.0247	0.9892
Antidepressant	0.7931	0.3366 *	0.9351	1.3572
Benzodiazepine	1.9445 *	2.3187	1.8857 *	1.6705 *
Calcium Channel Blocker	6.7194 **	3.4212	2.1308	3.297 *
Diabetes_2002	0.0000	6.4689	3.0649	4.1871
Diabetes_2003	0.0000	0.0000	0.0000	0.0000
Diabetes_2004	0.0000	0.0000	0.0000	0.0000
Diabetes_2005	4.7304	1.2090	0.0000	0.8411
Diabetes_2006	2.7319	8.602 **	2.7566	3.9118 *
Diabetes_2007	1.4627	2.8207	0.8961	1.4748
Diabetes_2008	0.8738	0.0000	0.6597	0.5084
Diabetes_2009	0.0000	0.0000	0.0000	0.0000
Diabetes_2010	0.0000	0.0000	0.0000	0.0000
Diabetes_2011	1.7329	0.0000	0.0000	4.5494 *
Diabetes_2012	1.1298	0.0000	0.8382	0.0000
Diabetes_2013	3.4178 *	0.0000	4.0511 *	2.9241
Diabetes_2014	0.0000	1.0787	0.8086	0.1108
Hypertension_2002	0.0000	0.0000	0.0000	0.0000
Hypertension_2003	0.0000	0.0000	M	0.0000
Hypertension_2004	0.0000	27.688 **	3.4349	3.0957
Hypertension_2005	0.0000	1.3907	0.0000	1.4404
Hypertension_2006	7.3522	M	M	5.5124
Hypertension_2007	0.4594	0.0000	0.0000	0.0000
Hypertension_2008	35.6946 **	69.4079	M	M
Hypertension_2009	12.9229 **	5.7489	4.3275	16.7433 **
Hypertension_2010	0.0000	0.0000	0.0000	0.1630
Hypertension_2011	2.0824	0.0000	3.2252	0.0000
Hypertension_2012	4.6089	0.0000	13.9992 *	2.1780
Hypertension_2013	2.2349	0.0000	0.2329	0.0000
Hypertension_2014	1.7902	0.0000	1.0933	0.0000
Myoma Uteri	0.5859	0.4173	1.1059	0.9930
Nitrate	40.813 **	0.0914	8.1989 **	7.0922 **
Prior Cone	8.2979 *	0.0000	13.5855 *	0.0000
Progesterone	1.1946	0.9761	1.2636	1.0069
Proton Pump Inhibitor	1.0064	2.3587 **	0.9217	0.9230
Region (City) in 2014	0.8083	0.4475	0.6042	0.6428
Salivary Gland_2002	0.2207	0.0000	0.0000	0.5387
Salivary Gland_2003	0.0000	0.0000	0.0000	0.0000
Salivary Gland_2004	0.0000	0.0601	0.0000	0.0000
Salivary Gland_2005	0.0000	0.0000	0.0000	0.0000
Salivary Gland_2006	0.0000	0.0000	0.0000	0.0000
Salivary Gland_2007	0.0000	4.7351	0.0000	1.4178
Salivary Gland_2008	0.0000	4.8404	0.0000	3.1393
Salivary Gland_2009	0.0000	0.0000	1.8714	1.9864
Salivary Gland_2010	5.8416	10.7941	2.6198	2.9447
Salivary Gland_2011	5.8416	0.0000	4.7588	1.4743
Salivary Gland_2012	6.8483	0.0000	0.0000	2.1834
Salivary Gland_2013	0.0000	0.0000	0.0000	0.0000
Salivary Gland_2014	26.1017 **	7.1278	3.2970	43.5539 **
Sleeping Pills	0.8594	1.0148	1.1157	0.6741
Socioeconomic Status	1.0009	0.9781	0.9951	0.9870

Note. ^a^ PTB Preterm Birth during 2015–2017. * *p < 0.10*. ** *p < 0.05*, *M: larger than 50.*

**Table 3:** Logistic Regression Results for Each Type of Preterm Birth: Crohn Group.

Determinant	*PTB 1* ^a^	*PTB 2*	*PTB 3*	*PTB 4*
Age	0.9612	0.9182	0.9529	0.9483
Antidepressant	1.4643	0.9280	1.2048	1.0858
Benzodiazepine	1.1167	0.5946	1.0122	0.9757
Calcium Channel Blocker	3.1582	1.2433	5.8533 **	2.2185
Diabetes_2002	0.0000	0.0000	0.0000	0.0000
Diabetes_2003	9.8355 *	0.0000	7.9486 *	19.4796 **
Diabetes_2004	0.0000	8.1336	3.0526	2.9233
Diabetes_2005	0.0000	5.3495	19.0678 **	2.3422
Diabetes_2006	0.0000	0.0000	0.0000	0.0000
Diabetes_2007	0.0000	0.0000	0.0000	0.0000
Diabetes_2008	0.0000	0.0000	0.0000	0.0000
Diabetes_2009	1.5868	8.3228 *	3.7584	0.9326
Diabetes_2010	0.0000	6.2651 *	0.0000	2.9998
Diabetes_2011	10.7725 *	7.0006	14.1682 **	3.3617
Diabetes_2012	0.0000	0.0000	0.0000	0.0000
Diabetes_2013	0.9761	0.0000	0.0000	0.0000
Diabetes_2014	0.9794	6.1411 **	0.6349	3.3018 *
Hypertension_2002	0.0000	0.0000	0.0000	0.0000
Hypertension_2003	0.0000	4.9828	7.5157	3.6272
Hypertension_2004	0.0000	4.1829	0.0000	1.9322
Hypertension_2005	2.0247	3.5431	1.6596	2.5593
Hypertension_2006	3.1614	0.0379	1.8278	1.1823
Hypertension_2007	0.0000	3.3974	0.0000	0.2921
Hypertension_2008	3.4834	1.5947	0.0000	0.5604
Hypertension_2009	7.5233 *	1.8647	5.1141	4.3998
Hypertension_2010	0.0000	0.8326	0.0000	1.8357
Hypertension_2011	0.0000	8.7583	0.0000	15.6653
Hypertension_2012	0.0000	0.9981	0.0000	0.1479
Hypertension_2013	0.0000	0.2764	0.0000	0.6883
Hypertension_2014	0.0000	4.8163	0.5982	1.6377
Myoma Uteri	0.7619	1.3336	1.5456	1.4259
Nitrate	2.0865	0.0000	1.8746	1.8284
Prior Cone	NA	0.0000	NA	0.0000
Progesterone	2.0049 **	1.8662	1.8207 **	2.2548 **
Proton Pump Inhibitor	0.7541	1.2072	1.1347	0.9458
Region (City) in 2014	1.2852	0.4652	0.5451 *	0.9668
Salivary Gland_2002	NA	0.0000	NA	0.0000
Salivary Gland_2003	0.0000	0.0000	0.0000	0.0000
Salivary Gland_2004	0.0000	0.0000	0.0000	0.0000
Salivary Gland_2005	0.9406	0.0000	189519030.0742	56.6879 **
Salivary Gland_2006	2.7456	0.0000	1.9636	1.4216
Salivary Gland_2007	0.0000	0.0000	0.0000	0.0000
Salivary Gland_2008	0.0000	0.0000	0.0000	0.0000
Salivary Gland_2009	0.0000	0.0000	0.0000	0.0000
Salivary Gland_2010	0.0000	0.0000	0.0000	11.644 **
Salivary Gland_2011	5.7892	0.0000	3.7886	3.9478
Salivary Gland_2012	0.0000	0.0000	0.0000	0.0000
Salivary Gland_2013	0.0000	0.0000	0.0000	0.0000
Salivary Gland_2014	0.0000	0.0000	0.0000	0.0000
Sleeping Pills	0.7012	1.1153	0.9745	0.6289
Socioeconomic Status	1.0056	1.0578	1.0169	0.9893

Note. ^a^ PTB Preterm Birth during 2015–2017. * *p < 0.10.* ** *p < 0.05*, *M: larger than 50.*

**Table 4:** Logistic Regression Results for Each Type of Preterm Birth: Non-Inflammatory Bowel Disease Group.

Determinant	*PTB 1* ^a^	*PTB 2*	*PTB 3*	*PTB 4*
Age	0.9864 **	0.9493 **	0.9812 **	0.9803 **
Antidepressant	1.1138 **	1.0266	1.0942 **	1.0697 **
Benzodiazepine	1.0398 **	1.0190	1.0379 **	1.0369 **
Calcium Channel Blocker	1.0493	1.2628 **	1.1522 *	1.1525 *
Diabetes_2002	1.1609	1.0846	1.1331	1.0639
Diabetes_2003	1.0182	1.1752	1.1132	1.2316
Diabetes_2004	1.0051	1.3507	0.9219	1.0140
Diabetes_2005	1.0878	1.0050	1.0904	1.1378
Diabetes_2006	1.1391	1.1016	1.1480	0.9728
Diabetes_2007	1.0264	1.0572	1.0508	1.0159
Diabetes_2008	1.1334	1.0637	1.0121	0.9703
Diabetes_2009	1.0951	1.2045	1.0927	1.1808 *
Diabetes_2010	1.1063	1.0970	1.0954	1.1200
Diabetes_2011	0.8018 *	0.9057	0.9544	0.9632
Diabetes_2012	1.1889 *	1.1856	1.1403	1.1347
Diabetes_2013	0.9975	1.0221	0.9668	1.0445
Diabetes_2014	1.1579 **	1.7234 **	1.4539 **	1.3475 **
Hypertension_2002	0.5747 **	0.7868	0.6063 **	0.6404 **
Hypertension_2003	1.1511	0.6140	1.2541	1.2604
Hypertension_2004	0.7358	0.8369	0.7422	0.6295 **
Hypertension_2005	1.0448	0.9915	0.9587	1.0616
Hypertension_2006	1.4625 **	1.1043	1.4599 **	1.3316 **
Hypertension_2007	1.0695	1.0451	0.9113	0.9915
Hypertension_2008	1.0781	1.2240	1.1505	1.0758
Hypertension_2009	0.9630	1.0183	1.0273	0.8976
Hypertension_2010	1.3628 **	1.0213	1.1403	1.317 **
Hypertension_2011	0.8217	0.8861	0.8902	1.0492
Hypertension_2012	0.9502	0.8388	0.9512	0.9595
Hypertension_2013	0.8954	1.4962 **	1.1539	1.1752
Hypertension_2014	1.2071	1.6816 **	1.3486 **	1.2994 **
Myoma Uteri	1.2391 **	1.5687 **	1.3566 **	1.3618 **
Nitrate	1.0039	1.1597	1.0187	1.1300
Prior Cone	2.0277 **	0.7387	1.2255	1.3424
Progesterone	1.2098 **	1.5060 **	1.2892 **	1.3095 **
Proton Pump Inhibitor	1.0587 **	0.9969	1.0481 **	1.0401 **
Region (City) in 2014	1.0875 **	0.8727 **	1.0417	1.0673 **
Salivary Gland_2002	1.1365	1.9245 **	1.0864	0.8633
Salivary Gland_2003	0.9571	0.6351	0.9949	1.0453
Salivary Gland_2004	0.8617	0.6470	0.5500 **	0.5602 **
Salivary Gland_2005	1.1713	1.1267	0.9054	1.0701
Salivary Gland_2006	1.2927	1.1820	1.4859 **	1.1396
Salivary Gland_2007	0.7483	0.9624	0.7269 *	0.8904
Salivary Gland_2008	1.0409	0.9801	0.9911	1.0023
Salivary Gland_2009	1.4634 **	1.1146	1.3290 **	1.2896 **
Salivary Gland_2010	0.6818 **	0.9678	0.9079	0.9039
Salivary Gland_2011	1.0066	0.9210	0.9264	1.0570
Salivary Gland_2012	0.9522	0.9528	0.9342	0.9912
Salivary Gland_2013	1.0885	0.8104	0.8957	0.9630
Salivary Gland_2014	1.0097	0.5847 **	0.9340	0.9446
Sleeping Pills	0.9502	1.0534	0.9805	0.9992
Socioeconomic Status	1.0020	1.0043	1.0027 *	1.0022

Note. ^a^ PTB Preterm Birth during 2015–2017, * *p < 0.10*, ** *p < 0.05*, *M: larger than 50.*

## Data Availability

The data presented in this study are not publicly available. However, the data are available from the corresponding author upon reasonable request and under the permission of Korea National Health Insurance Service.

## Supplementary Materials

The following are available online at https://www.mdpi.com/article/10.3390/ijerph19053056/s1, Table S1: ICD-10 Code for Preterm Birth, Inflammatory Bowel Disease and Salivary Gland Disease; Table S2: ATC Code for Medication; Table S3: Descriptive Statistics on Preterm Birth and Its Determinants of All; Table S4: Descriptive Statistics on Preterm Birth and Its Determinants of Ulcerative-Colitis Group; Table S5: Descriptive Statistics on Preterm Birth and Its Determinants of Crohn Group; Table S6: Descriptive Statistics on Preterm Birth and Its Determinants of Non-Inflammatory-Bowel-Disease Group; Figure S1: Average Values of Random Forest Variable Importance for PTB 1, PTB 2, PTB 3 and PTB 4: Ulcerative-Colitis Group; Figure S2: Average Values of Random Forest Variable Importance for PTB 1, PTB 2, PTB 3 and PTB 4: Crohn Group; Figure S3: Average Values of Random Forest Variable Importance for PTB 1, PTB 2, PTB 3 and PTB 4: Non-Inflammatory-Bowel-Disease Group.
