# Supplementary material for: Association of Preterm Birth with Inflammatory Bowel Disease and Salivary Gland Disease: Machine Learning Analysis Using National Health Insurance Data
Source: Int J Environ Res Public Health. 2022 Mar 5;19(5):3056. doi: 10.3390/ijerph19053056 (PMC8910052; doi:10.3390/ijerph19053056)

Table S1. ICD-10 Code for Preterm Birth, Inflammatory Bowel Disease and Salivary Gland Disease.

		**Code**	**Description**
PTB	PROM	O42.00	Onset of labor within 24 hours of rupture (0-33 weeks of gestation)
		O42.01	Onset of labor within 24 hours of rupture (34-36 weeks of gestation)
		O42.10	Onset of labor after 24 hours of rupture (0-33 weeks of gestation)
		O42.11	Onset of labor after 24 hours of rupture (34-36 weeks of gestation)
		O42.20	Prolonged labor because of treatment (0-33 weeks of gestation)
		O42.21	Prolonged labor because of treatment (34-36 weeks of gestation)
		O42.90	Unspecified length of time between rupture and onset of labor (0-33 weeks of gestation)
		O42.91	Unspecified length of time between rupture and onset of labor (34-36 weeks of gestation)
	PTL	O60.11	Preterm delivery with preterm labor (0-33 weeks of gestation)
		O60.12	Preterm delivery with preterm labor (34-36 weeks of gestation)
		O60.19	Preterm delivery with preterm labor (unspecified weeks of gestation)
	Other	O60.31	Preterm delivery without preterm labor (0-33 weeks of gestation)
		O60.32	Preterm delivery without preterm labor (34-36 weeks of gestation)
		O60.39	Preterm delivery without preterm labor (unspecified weeks of gestation)
Crohn		K50	Crohn’s disease
UC		K51	Ulcerative colitis
SG		K11	Salivary gland disease

Abbreviations: PTB Preterm Birth, PROM Premature Rupture of Membranes, PTL Preterm Labor with Preterm Delivery, UC Ulcerative Colitis, SG Salivary Gland Disease

Table S2. ATC Code for Medication.

Medication	Code	Description	
Antidepressant	N06A	Antidepressants	
Benzodiazepine	N05BA	Benzodiazepine derivatives	(N05 Psychoanaleptics, N05B Anxiolytics)
	N05CD	Benzodiazepine derivatives	(N05 Psychoanaleptics, N05C Hypnotics and sedatives)
	N05CF	Benzodiazepine-related drugs	(N05 Psychoanaleptics, N05C Hypnotics and sedatives)
Calcium Channel Blocker	C08	Calcium channel blockers	
Nitrate	C01DA	Organic nitrates	
Progesterone	G03	Sex hormones and modulators of the genital system	
Proton Pump Inhibitor	A02BC	Proton pump inhibitors	
Sleeping Pills	N05C	Hypnotics and sedatives	

Table S3. Descriptive Statistics on Preterm Birth and Its Determinants of All.

**Variable**	*No*	*Yes*	*Yes (%)*
PTB 1[a]	380231	21724	5.40
PTB 2	393030	8925	2.22
PTB 3	374212	27743	6.90
PTB 4	373120	28835	7.17
Antidepressant	338934	63021	15.68
Benzodiazepine	165738	236217	58.77
Calcium Channel Blocker	398218	3737	0.93
Crohn's Disease_2002	401545	410	0.10
Crohn's Disease_2003	401654	301	0.07
Crohn's Disease_2004	401649	306	0.08
Crohn's Disease_2005	401697	258	0.06
Crohn's Disease_2006	401776	179	0.04
Crohn's Disease_2007	401825	130	0.03
Crohn's Disease_2008	401862	93	0.02
Crohn's Disease_2009	401858	97	0.02
Crohn's Disease_2010	401851	104	0.03
Crohn's Disease_2011	401837	118	0.03
Crohn's Disease_2012	401842	113	0.03
Crohn's Disease_2013	401840	115	0.03
Crohn's Disease_2014	401834	121	0.03
Diabetes_2002	401091	864	0.21
Diabetes_2003	400946	1009	0.25
Diabetes_2004	400698	1257	0.31
Diabetes_2005	400172	1783	0.44
Diabetes_2006	400212	1743	0.43
Diabetes_2007	400168	1787	0.44
Diabetes_2008	400076	1879	0.47
Diabetes_2009	399927	2028	0.50
Diabetes_2010	399699	2256	0.56
Diabetes_2011	399356	2599	0.65
Diabetes_2012	398893	3062	0.76
Diabetes_2013	397914	4041	1.01
Diabetes_2014	395569	6386	1.59
Hypertension_2002	401356	599	0.15
Hypertension_2003	401327	628	0.16
Hypertension_2004	401223	732	0.18
Hypertension_2005	401060	895	0.22
Hypertension_2006	400951	1004	0.25
Hypertension_2007	400831	1124	0.28
Hypertension_2008	400707	1248	0.31
Hypertension_2009	400581	1374	0.34
Hypertension_2010	400578	1377	0.34
Hypertension_2011	400601	1354	0.34
Hypertension_2012	400269	1686	0.42
Hypertension_2013	400052	1903	0.47

Hypertension_2014	399716	2239	0.56
Myoma Uteri	384884	17071	4.25
Nitrate	400640	1315	0.33
Prior Cone	401774	181	0.05
Progesterone	307590	94365	23.48
Proton Pump Inhibitor	276019	125936	31.33
Region (City) in 2014	28602	373353	92.88
Salivary Gland_2002	401612	343	0.09
Salivary Gland_2003	401475	480	0.12
Salivary Gland_2004	401368	587	0.15
Salivary Gland_2005	401317	638	0.16
Salivary Gland_2006	401184	771	0.19
Salivary Gland_2007	401074	881	0.22
Salivary Gland_2008	401008	947	0.24
Salivary Gland_2009	400713	1242	0.31
Salivary Gland_2010	400728	1227	0.31
Salivary Gland_2011	400572	1383	0.34
Salivary Gland_2012	400460	1495	0.37
Salivary Gland_2013	400318	1637	0.41
Salivary Gland_2014	400202	1753	0.44
Sleeping Pills	370188	31767	7.90
Ulcerative Colitis_2002	401793	162	0.04
Ulcerative Colitis_2003	401750	205	0.05
Ulcerative Colitis_2004	401777	178	0.04
Ulcerative Colitis_2005	401761	194	0.05
Ulcerative Colitis_2006	401786	169	0.04
Ulcerative Colitis_2007	401769	186	0.05
Ulcerative Colitis_2008	401760	195	0.05
Ulcerative Colitis_2009	401747	208	0.05
Ulcerative Colitis_2010	401718	237	0.06
Ulcerative Colitis_2011	401714	241	0.06
Ulcerative Colitis_2012	401690	265	0.07
Ulcerative Colitis_2013	401654	301	0.07
Ulcerative Colitis_2014	401631	324	0.08

Note: [a] PTB Preterm Birth during 2015-2017

	**Min**	**Q1**	**Median**	**Q3**	**Max**
Age in 2014	22	27	29	32	39
Socioeconomic Status in 2014	1	8	12	15	20

Table S4. Descriptive Statistics on Preterm Birth and Its Determinants of Ulcerative-Colitis Group.

**Variable**	*No*	*Yes*	*Yes (%)*
PTB 1[a]	1677	105	5.89
PTB 2	1737	45	2.53
PTB 3	1648	134	7.52
PTB 4	1642	140	7.86
Antidepressant	1336	446	25.03
Benzodiazepine	431	1351	75.81
Calcium Channel Blocker	1754	28	1.57
Diabetes_2002	1774	8	0.45
Diabetes_2003	1776	6	0.34
Diabetes_2004	1772	10	0.56
Diabetes_2005	1768	14	0.79
Diabetes_2006	1767	15	0.84
Diabetes_2007	1762	20	1.12
Diabetes_2008	1769	13	0.73
Diabetes_2009	1769	13	0.73
Diabetes_2010	1773	9	0.51
Diabetes_2011	1763	19	1.07
Diabetes_2012	1758	24	1.35
Diabetes_2013	1751	31	1.74
Diabetes_2014	1736	46	2.58
Hypertension_2002	1776	6	0.34
Hypertension_2003	1778	4	0.22
Hypertension_2004	1776	6	0.34
Hypertension_2005	1777	5	0.28
Hypertension_2006	1779	3	0.17
Hypertension_2007	1773	9	0.51
Hypertension_2008	1776	6	0.34
Hypertension_2009	1769	13	0.73
Hypertension_2010	1773	9	0.51
Hypertension_2011	1774	8	0.45
Hypertension_2012	1767	15	0.84
Hypertension_2013	1771	11	0.62
Hypertension_2014	1768	14	0.79
Myoma Uteri	1688	94	5.27
Nitrate	1773	9	0.51
Prior Cone	1779	3	0.17
Progesterone	1220	562	31.54
Proton Pump Inhibitor	941	841	47.19
Region (City) in 2014	138	1644	92.26
Salivary Gland_2002	1779	3	0.17
Salivary Gland_2003	1776	6	0.34
Salivary Gland_2004	1777	5	0.28
Salivary Gland_2005	1775	7	0.39
Salivary Gland_2006	1779	3	0.17
Salivary Gland_2007	1776	6	0.34

Salivary Gland_2008	1773	9	0.51
Salivary Gland_2009	1775	7	0.39
Salivary Gland_2010	1774	8	0.45
Salivary Gland_2011	1775	7	0.39
Salivary Gland_2012	1774	8	0.45
Salivary Gland_2013	1773	9	0.51
Salivary Gland_2014	1774	8	0.45
Sleeping Pills	1552	230	12.91

Note: [a] PTB Preterm Birth during 2015-2017

	**Min**	**Q1**	**Median**	**Q3**	**Max**
Age in 2014	22	27	30	33	39
Socioeconomic Status in 2014	1	9	12	15	20

Table S5. Descriptive Statistics on Preterm Birth and Its Determinants of Crohn Group.

**Variable**	*No*	*Yes*	*Yes (%)*
PTB 1[a]	1863	91	4.66
PTB 2	1908	46	2.35
PTB 3	1825	129	6.60
PTB 4	1822	132	6.76
Antidepressant	1465	489	25.03
Benzodiazepine	465	1489	76.20
Calcium Channel Blocker	1925	29	1.48
Diabetes_2002	1949	5	0.26
Diabetes_2003	1946	8	0.41
Diabetes_2004	1943	11	0.56
Diabetes_2005	1938	16	0.82
Diabetes_2006	1947	7	0.36
Diabetes_2007	1946	8	0.41
Diabetes_2008	1938	16	0.82
Diabetes_2009	1933	21	1.07
Diabetes_2010	1935	19	0.97
Diabetes_2011	1938	16	0.82
Diabetes_2012	1925	29	1.48
Diabetes_2013	1924	30	1.54
Diabetes_2014	1920	34	1.74
Hypertension_2002	1948	6	0.31
Hypertension_2003	1946	8	0.41
Hypertension_2004	1949	5	0.26
Hypertension_2005	1944	10	0.51
Hypertension_2006	1941	13	0.67
Hypertension_2007	1947	7	0.36
Hypertension_2008	1944	10	0.51
Hypertension_2009	1942	12	0.61
Hypertension_2010	1942	12	0.61
Hypertension_2011	1945	9	0.46
Hypertension_2012	1938	16	0.82
Hypertension_2013	1937	17	0.87
Hypertension_2014	1939	15	0.77
Myoma Uteri	1848	106	5.42
Nitrate	1938	16	0.82
Prior Cone	1953	1	0.05
Progesterone	1378	576	29.48
Proton Pump Inhibitor	1037	917	46.93
Region (City) in 2014	165	1789	91.56
Salivary Gland_2002	1952	2	0.10
Salivary Gland_2003	1953	1	0.05
Salivary Gland_2004	1952	2	0.10
Salivary Gland_2005	1948	6	0.31
Salivary Gland_2006	1948	6	0.31
Salivary Gland_2007	1947	7	0.36

Salivary Gland_2008	1948	6	0.31
Salivary Gland_2009	1948	6	0.31
Salivary Gland_2010	1948	6	0.31
Salivary Gland_2011	1945	9	0.46
Salivary Gland_2012	1945	9	0.46
Salivary Gland_2013	1944	10	0.51
Salivary Gland_2014	1943	11	0.56
Sleeping Pills	1705	249	12.74

Note: [a] PTB Preterm Birth during 2015-2017

	Min	Q1	Median	Q3	Max
Age in 2014	22	27	30	32	39
Socioeconomic Status in 2014	1	8	12	15	20

Table S6. Descriptive Statistics on Preterm Birth and Its Determinants of Non-Inflammatory-Bowel-Disease Group.

**Variable**	*No*	*Yes*	*Yes (%)*
PTB 1[a]	376691	21528	5.41
PTB 2	389385	8834	2.22
PTB 3	370739	27480	6.90
PTB 4	369656	28563	7.17
Antidepressant	336133	62086	15.59
Benzodiazepine	164842	233377	58.61
Calcium Channel Blocker	394539	3680	0.92
Diabetes_2002	397368	851	0.21
Diabetes_2003	397224	995	0.25
Diabetes_2004	396983	1236	0.31
Diabetes_2005	396466	1753	0.44
Diabetes_2006	396498	1721	0.43
Diabetes_2007	396460	1759	0.44
Diabetes_2008	396369	1850	0.46
Diabetes_2009	396225	1994	0.50
Diabetes_2010	395991	2228	0.56
Diabetes_2011	395655	2564	0.64
Diabetes_2012	395210	3009	0.76
Diabetes_2013	394239	3980	1.00
Diabetes_2014	391913	6306	1.58
Hypertension_2002	397632	587	0.15
Hypertension_2003	397603	616	0.15
Hypertension_2004	397498	721	0.18
Hypertension_2005	397339	880	0.22
Hypertension_2006	397231	988	0.25
Hypertension_2007	397111	1108	0.28
Hypertension_2008	396987	1232	0.31
Hypertension_2009	396870	1349	0.34
Hypertension_2010	396863	1356	0.34
Hypertension_2011	396882	1337	0.34
Hypertension_2012	396564	1655	0.42
Hypertension_2013	396344	1875	0.47
Hypertension_2014	396009	2210	0.55
Myoma Uteri	381348	16871	4.24
Nitrate	396929	1290	0.32
Prior Cone	398042	177	0.04
Progesterone	304992	93227	23.41
Proton Pump Inhibitor	274041	124178	31.18
Region (City) in 2014	28299	369920	92.89
Salivary Gland_2002	397881	338	0.08
Salivary Gland_2003	397746	473	0.12
Salivary Gland_2004	397639	580	0.15
Salivary Gland_2005	397594	625	0.16
Salivary Gland_2006	397457	762	0.19

Salivary Gland_2007	397351	868	0.22
Salivary Gland_2008	397287	932	0.23
Salivary Gland_2009	396990	1229	0.31
Salivary Gland_2010	397006	1213	0.30
Salivary Gland_2011	396852	1367	0.34
Salivary Gland_2012	396741	1478	0.37
Salivary Gland_2013	396601	1618	0.41
Salivary Gland_2014	396485	1734	0.44
Sleeping Pills	366931	31288	7.86

Note: [a] PTB Preterm Birth during 2015-2017

	**Min**	**Q1**	**Median**	**Q3**	**Max**
Age in 2014	22	27	29	32	39
Socioeconomic Status in 2014	1	8	12	15	20

Figure S1. Average Values of Random Forest Variable Importance for PTB 1, PTB 2, PTB 3 and PTB 4: Ulcerative-Colitis Group.

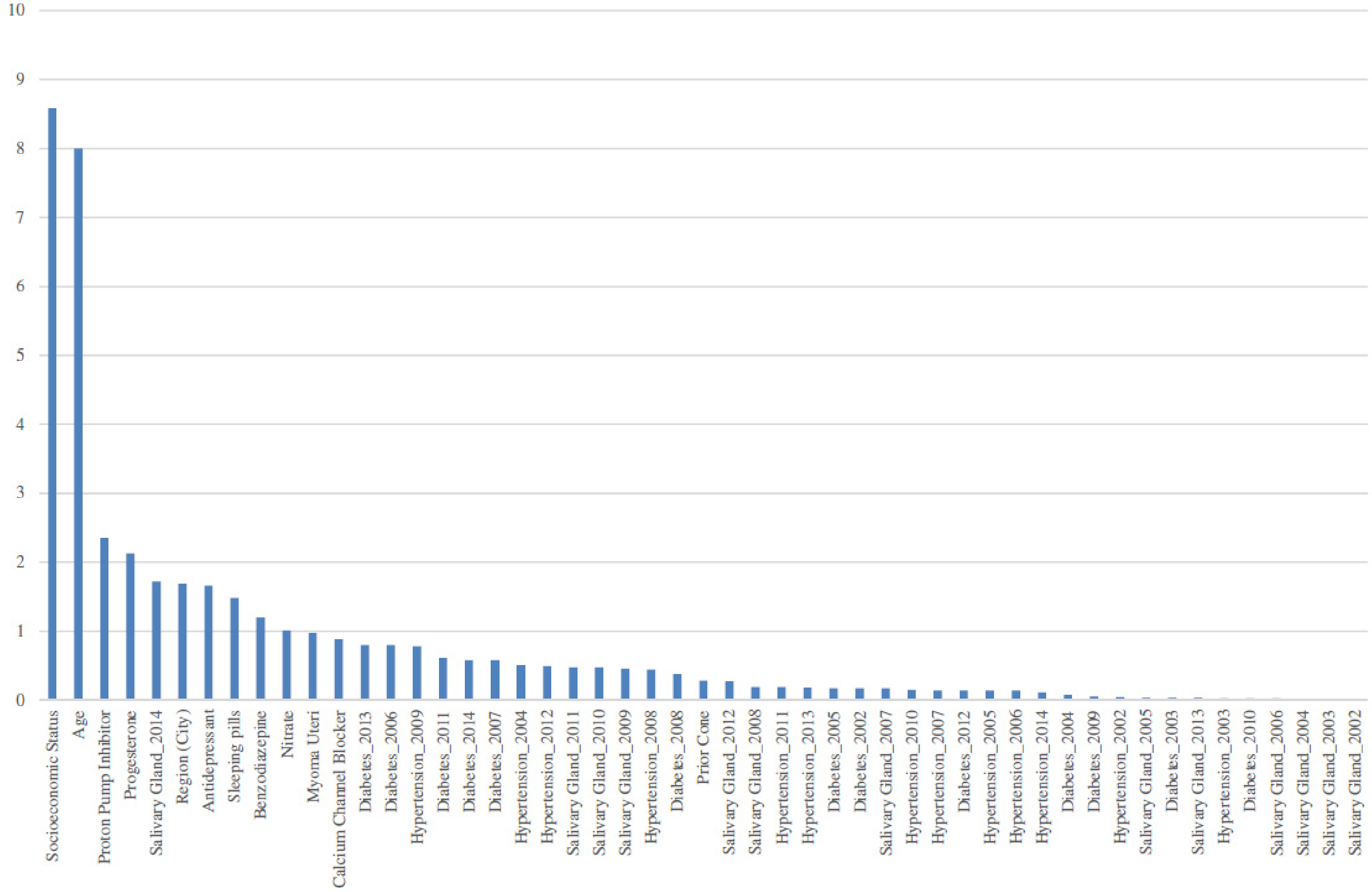

Figure S2. Average Values of Random Forest Variable Importance for PTB 1, PTB 2, PTB 3 and PTB 4: Crohn Group.

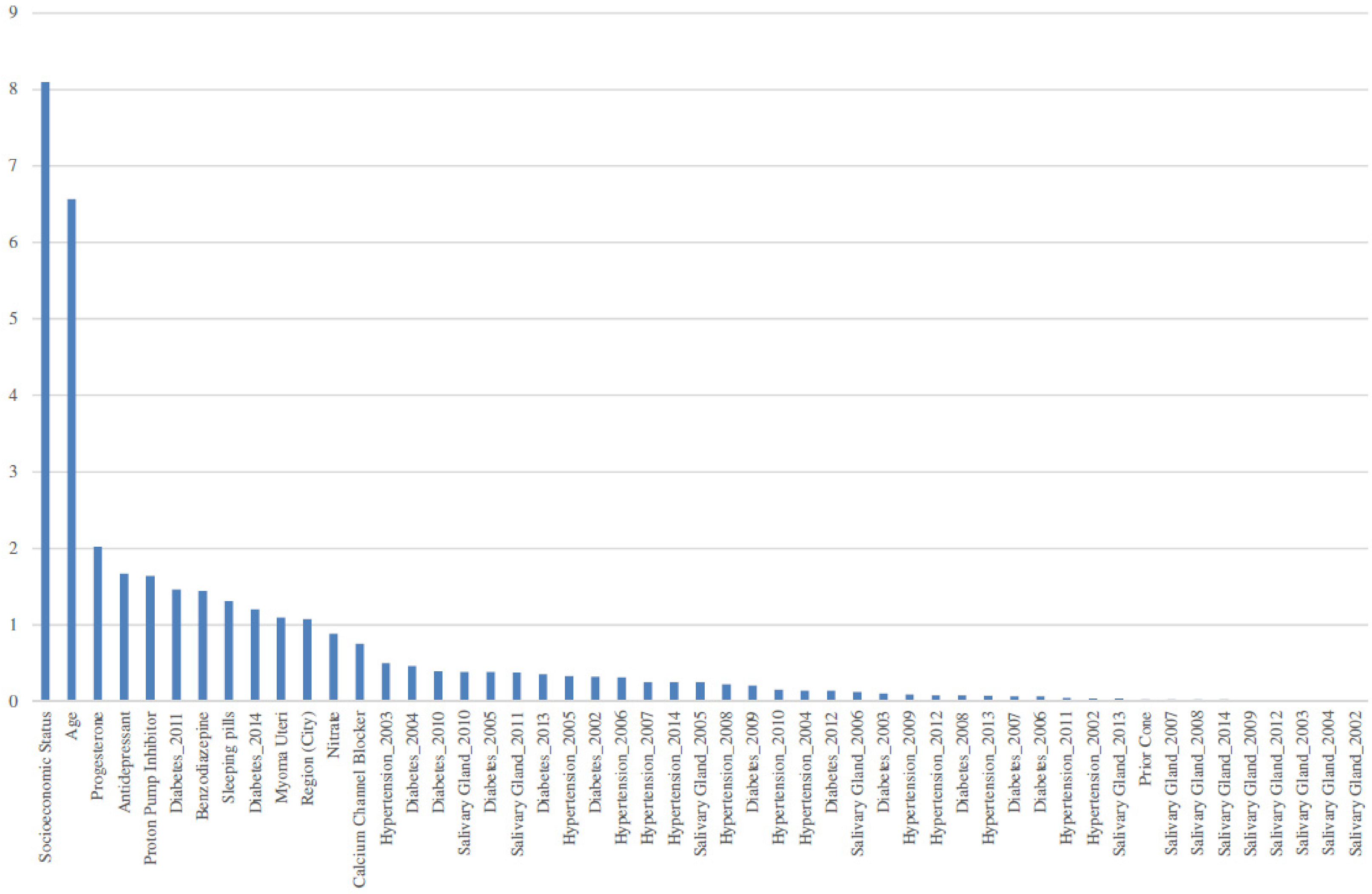

Figure S3. Average Values of Random Forest Variable Importance for PTB 1, PTB 2, PTB 3 and PTB 4: Non-Inflammatory-Bowel-Disease Group.

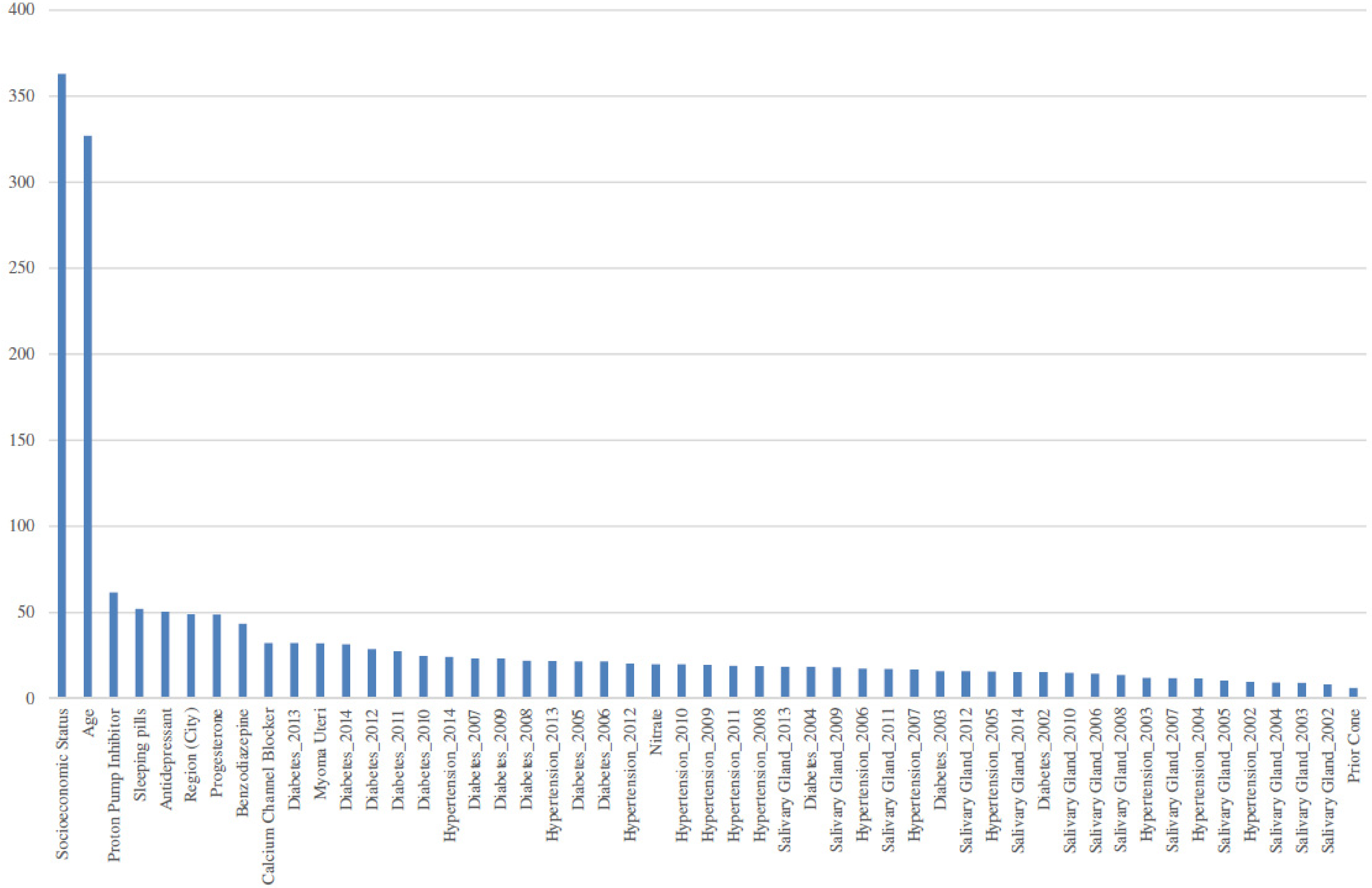

Supplement: Supplementary file 1 [file ijerph-19-03056-s001.zip › ijerph-1572296-supplementary.pdf]
